# A 2-Week Specific Volleyball Training Supported by the Whole Body Cryostimulation Protocol Induced an Increase of Growth Factors and Counteracted Deterioration of Physical Performance

**DOI:** 10.3389/fphys.2018.01711

**Published:** 2018-11-28

**Authors:** Joanna Jaworska, Katarzyna Micielska, Marta Kozłowska, Krzysztof Wnorowski, Jerzy Skrobecki, Lukasz Radzimiński, Anna Babińska, Ewa Rodziewicz, Giovanni Lombardi, Ewa Ziemann

**Affiliations:** ^1^Department of Physiology and Pharmacology, Faculty of Rehabilitation and Kinesiology, Gdansk University of Physical Education and Sport, Gdańsk, Poland; ^2^Department of Anatomy and Anthropology, Faculty of Physical Education, Gdansk University of Physical Education and Sport, Gdańsk, Poland; ^3^Department of Volleyball, Faculty of Physical Education, Gdansk University of Physical Education and Sport, Gdańsk, Poland; ^4^Department of Biomedical Health Basics, Faculty of Tourism and Recreation, Gdansk University of Physical Education and Sport, Gdańsk, Poland; ^5^Department of Endocrinology and Internal Medicine, Medical University, Gdańsk, Poland; ^6^Laboratory of Experimental Biochemistry and Molecular Biology, IRCCS Istituto Ortopedico Galeazzi, Milan, Italy

**Keywords:** myokines, training adaptations, branched amino acids, tryptophan, coldness

## Abstract

Potentially beneficial effects of cold therapies on training adaptation still remain unequivocal. We have, thus, decided to evaluate the effects of a 2-week volleyball training program supported by 10 sessions of whole body cryostimulation (WBC) on growth factors and physical performance. Twenty healthy college-aged men and women randomly assigned either to the cryostimulation group (CRY) or the control group (CON; executed passive rest). Both groups took part in the same 2-weeks training program. Additionally, the CRY group attended in 10 cryo-sessions (3 min, -110°C temperature, five times/week). Blood samples were collected at baseline, 1 h after the first cryo-session as well as before and 1 h after the last session of WBC to assess growth factors, myokines concentration and the amino acid profile. Motor abilities were tested before commencing the training program and 2 days after its completion. The applied intervention resulted in an increase of brain-derived neurotrophic factor and insulin-like growth factor 1 concentrations. The adjusted effect describing the difference between groups in response to applied procedures was for both growth factors large and very likely in the CRY, higher than in the CON group (113%; Coefficient Interval: 38–230%, 45%; Coefficient Interval: 17–79%, respectively). Physical performance dropped in both groups, yet in the CRY group, the magnitude of change was smaller. The fibroblast growth factor dropped significantly 1 h following the first cryo-session, yet irisin remained statistically unchanged. The similar tendency was maintained after the whole procedure, still the range of changes was smaller. In the CRY group, an elevated uptake of tryptophan and valine noted in response to the whole intervention, could have induced a significant decrease of fasting glucose concentration (the adjusted effect small and very likely -6%; Coefficient Interval: -10 to -2%). Overall, a 2-week volleyball training program supported by the whole body cryostimulation protocol resulted in an increase of growth factors and offset a decline of physical performance. Thus these procedure can be applied in professional sport during competition period, especially among those disciplines focusing on an explosive power and ability to concentrate.

## Introduction

Different studies have demonstrated the effectiveness of whole body cryostimulation (WBC) in supporting recovery processes. This procedure can be applied in the preparation phase of training (Wozniak et al., 2007), in the midseason break/regenerative period (Ziemann et al., 2012) and after the competition season (Selfe et al., 2014). As it was shown to lower the perceived level of fatigue, WBC can be a useful way to counteract the onset of the overreaching state (Schaal et al., 2015). Generally, WBC treatments involve a 3-min exposure in a special cryo-chamber set at -110 to -140°C, depending on the cooling system (electrical or nitrogen). Such an exposure repeated 10 times has been shown to be sufficient to induce the anti-inflammatory response (Lombardi et al., 2017).

WBC is often compared with cold water immersion (CWI) treatment. Changes in muscle and core temperatures induced by these two cold therapies may indeed appear mostly similar, though not for the skin (Costello et al., 2012). The response of these therapies is depended on an applied kind of exercise, its duration, frequency, intensity and other factors: such body composition, gender and environmental conditions (Costello et al., 2015; Stephens et al., 2017). Recently published papers revealed that these two cold therapies used after high-intensity exercise, may not always bring significant and beneficial changes in muscle damage markers compare to other recovery methods (Chan et al., 2016; Krueger et al., 2018). Still, the review written by Rose and colleagues has shown that the application of WBC following an intensive exercise had an analgesic effect and improved physical performance in 71% of the analyzed studies. (Rose et al., 2017) However, regular post-exercise CWI (10°C, 10 min) has been demonstrated to inhibit muscular adaptation when following a strength training program (12 weeks, twice a week). Authors have observed that training supported by CWI attenuated acute changes in satellite cell numbers and the activity of kinases that regulate muscle hypertrophy (Roberts et al., 2015). Consequently, a possibility that the effect of a cold treatment may not necessarily be beneficial for training adaptation, it should be considered, as it may weaken the anabolic response triggered by resistance training.

Extensive metabolic adaptations in skeletal muscle are induced through various molecular pathways and myokines (interleukins: IL-6, IL-10, IL-15, irisin, and others), which not only act on muscles via an autocrine/paracrine manner, but also mediate interaction of muscles with other organs through endocrine mechanisms (Egan and Zierath, 2013) The influence of physical training on myokines’ concentrations is well documented, but data regarding the impact of cold treatments on their levels are still limited. The levels of irisin and fibroblast growth factor 21 (FGF21) have been shown to have shifted in response to cold exposure, leading to fat browning (Lee et al., 2014). At the same time, irisin changes have also been observed in response to resistance exercise (Knaepen et al., 2010) as well as after endurance training (Nygaard et al., 2015). It has also been revealed that irisin is crucial for training adaptation (Fatouros, 2018), while FGF21 predicts non-shivering thermogenesis response in humans (Lee et al., 2014). What is more, irisin can play a significant role in stimulating brain-derived neurotrophic factor (BDNF) (Zsuga et al., 2016) and this increase can affect human cognitive functions and their performance (Phillips et al., 2014). Among other myokines, which can modify an increase of anabolic signal in response to resistance training are interleukin 15 (IL-15) (Nielsen et al., 2007), insulin-like growth factor (IGF-1) (Fink et al., 2017) and myostatin (Huh, 2018).

Also, supplements (for example branched amino-acids/BCAA/), sometimes referred to as immuno-nutrients, often used in combination with resistance training, may reduce immunosuppression and excessive inflammation, regulating the anabolic signal as a result. The anabolic response to training is, thus, very complex as it involves adaptation over different levels. Importantly, this response is not limited to the skeletal muscle tissue, but depends on the integration of signals coming from the organs deputed to energy management, the immune system and the brain. Hence, the study of the adaptive response to training (i.e., damage, regeneration, anabolism) requires for at least the key factors involved in this crosstalk to be investigated as well (Giudice and Taylor, 2017). This is even more valid in the case when training (resistance) is combined with WBC; the combination which is known to induce whole-body homeostatic responses that somehow mimic the effects of exercise (Lombardi et al., 2017). Still, the influence of resistance training applied in conjunction with cold exposure on the anabolic response remains uncertain.

In light of the presented reasoning, our study was set out to investigate whether a combination of a 2-week resistance training program and 10 sessions of whole body cryostimulation would improve motor abilities and delay mental fatigue in college volleyball players. We have evaluated the effect of these two factors (training and cold exposure) on myokines’ levels and amino acids concentrations, due to their crucial role in training adaptation.

## Materials and Methods

### Study Design

**FIGURE 1:**
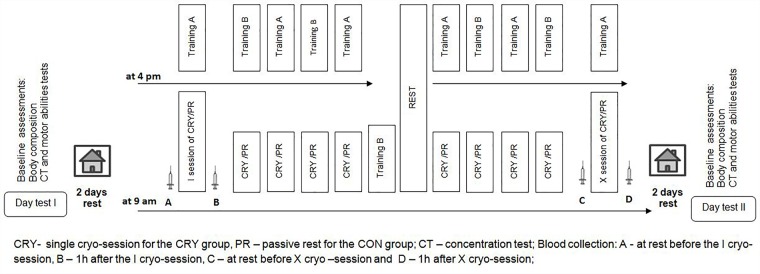
Experiment schedule.

The study design and timeline is presented in Figure 1. Two days before the main training program we performed a battery of tests. The same battery of test was repeated 2 days after the program’s completion.

### Subjects

A group of players from the university volleyball team took part in our experiment (*n* = 10 women, *n* = 10 men). The physical testing was performed before the randomization of participants into the subgroups. In order to avoid any effect on the final results, group allocation was done after the first physical performance assessment. The athletes were randomly assigned either to the experimental group (CRY; *n* = 8) or the control group (CON; *n* = 12). Two subjects in the CRY group had requested reallocation before the first WBC session due to hypersensitivity to low temperatures. With the two subjects transferred to the CON group, the groups became disproportionate. Subjects from the CON group did not participate in cryostimulation at any point of our study. In addition to basic parameters, the phase of menstrual cycles of female players was checked. We found that 70% of participating women were in the follicular phase, whereas the remaining female participants were in the luteal phase. Both groups participated in the same 2-week training program. All subjects lived in the same accommodation and followed the same training schedule and diet. Daily, energetic value of food offered in the menu did not exceed 3,800 kcal. The proposed protein dose varied from 1.2 to 1.4 g/kg of body mass (BM). Participants did not take any supplementation or prescription drugs during the study. The subjects were fully informed of the risks and stresses associated with the study and gave their written consent to participate.

### Anthropometric Measurements

Body mass and body composition were assessed before the start of the training program and 2 days after the intervention, using a multi-frequency impedance plethysmograph body composition analyzer (In Body 720, Biospace Analyzer, Korea). This analyzer accurately measures the amount of body water and body composition, including fat mass, free fat mass, skeletal muscle mass and soft lean mass.

### Physical and Mental Performance Assessment

Participants performed a 15-min standardized warm-up, including running, particular volleyball exercises and dynamic stretching exercises of upper and lower limbs. Afterward, athletes completed tests in the following order: a concentration test, explosive power assessment, a serve accuracy test, a single high-intensity interval bout of exercise and directly after its finishing the concentration test was repeated.

### Concentration Test

Grid exercise was applied to evaluate concentration and focus abilities. The original grid exercise uses a 10-by-10 block. Each block contains a two-digit number ranging from 00 to 99. The aim of this exercise is to scan the grid, and within a given a time period, find and click (cross-out) as many numbers as possible in an ascending numerical sequence (Wilson et al., 2006). In the present study, participants had 2 min to connect numbers from 00 to 99. The test was performed twice, at rest and directly after interval volleyball exercise. A time countdown was visible during the exercise to build up the pressure experienced by each subject.

### Explosive Power Measurements Test

Explosive power assessment consisted of series of countermovement jumps in two positions: a series of three countermovement jumps with arms rested on the hips and a series of three countermovement jumps with an arm swing with the shortest possible contact time between the feet and the surface. Subjects took a 3-min break between series. All jumps were performed on a tensometric mat (Smart Jump Mat 120 cm × 120 cm—Fusion Sport, Cooper Plains, Australia).

### Serve Accuracy Test

Serve accuracy was tested using Service Accuracy in resting conditions described by Lidor and colleagues. Players had to hit 10 consecutive serves into designated areas on the opposite side of the court (Lidor et al., 2007).

#### Interval Training Bout of Exercise

In order to induce fatigue similar to the one experienced during a professional match, a single high-intensity interval bout of exercise was performed. We decided to apply the work to rest ratio of 1:3 (10 s work and 30 s rest, repeated six times). We used a system of four double photoelectric cells (SMART SPEED PRO—Fusion Sport). We placed these cells round and opposite the player in the set-up of four gates. Subjects had to cross each lit up gate as soon as possible. The cells lit up randomly. Athletes began the test with one foot on the line in a standing starting position. All participants received standardized verbal instructions before the exercise and verbal encouragement throughout. Given the random and unrepeatable nature of the each test’s sequence, we did not compare the results and reaction time achieved in this test.

### Training Program

At the onset of the training program, a briefing session was organized to familiarize all participants with planned exercises. During the 2-week program, training was conducted once a day (including Sundays) with the total of 11 sessions. Players participated in six power training sessions at the gym (∼60 min per session) and five volleyball training sessions at the sports hall (∼120 min per session). Training details are presented in Table 1 and Figure 1. Each power training session also included a 10-min warm-up and 10 min of plyometric exercises. The volleyball training focused on discipline-specific performance. Participants worked on improving technical and tactical skills such as blocking, attacking, serving in simulated volleyball matches. All training sessions were supervised by players’ coaches.

**Table 1:** Characteristics of training sessions.

Training	Number of sessions	Time (min)	Load
Volleyball	5	120	Volleyball training improved technical and tactical performers: fife actions: serve, reception, setting, spike, block; high-intensity intervals 2:1 (8 min work and 4 min break) 85–90% MHR; 60 min Small games (simulated volleyball matches): 4 × 12 min games (with 4 min break); high intensity 90–95% MHR; 60 min
Power	6	60	Power training at the gym; six exercises for volleyball players, each at 35% of 1 RM, involving arms and legs muscles; three sets of six repetitions of: clean and jerk, snatch, explosive bench press, continuous squat jump (with barbell on the back), bound, split squat jump alternating; the break between sets lasted >2 min; each session was preceded 10 min of warm-up and 10 min of plyometric exercises

*MHR, maximal heart rate; RM, repetition maximum*.

### Whole-Body Cryostimulation

Along with following the training program, the CRY group attended 10 sessions of whole-body cryotherapy in a cryogenic chamber at the Pomeranian Rheumatologic Center in Sopot, Poland. Each cryo-session lasted 3 min at a temperature of -110°C. The procedure of this cold exposure was similar to the one commonly recommended and applied by our research team (Ziemann et al., 2014). The control group followed only the training program without using any recovery methods.

### Blood Collection and Analysis

Blood samples were taken by professional medical staff from the antecubital vein into vacutainer tubes with K2EDTA (Becton Dickinson & Co., Franklin Lakes, NJ, United States) on the first and last day of the training program, before breakfast and 1 h after the cryo-session. Immediately following blood collection, one sample was transferred into a centrifuge tube with aprotinin to undergo further irisin level assessment. We used tubes from Phoenix Pharmaceuticals Inc. (catalog no RK-APRO). The final concentration of aprotinin was 0.6 Trypsin Inhibitor Unit/1 mL of blood. The samples were centrifuged at 2,000 ×*g* for 10 min at 4°C and the serum was stored at -80°C until later analysis. Concentrations of serum IL-15, IL-6, myostatin and BDNF were assessed using sandwich ELISA kit according to the manufacturers’ instructions (R&D Systems, United States, catalog no D1500, HS600B, DGDF80 and DBD00, respectively). The average intra-assay coefficient of variability (CV) for IL-15, myostatin and BDNF was 5%. The inter-assay coefficient and detection sensitivity were as follows: 9.1% and 2 pg mL^-1^ for IL-15; 6% and 5.32 pg mL^-1^ for myostatin and 11.3% and 20 pg mL^-1^ for BDNF. The detection limit for BDNF was <20 pg mL^-1^ and 2.25 pg mL^-1^ for myostatin. The samples of myostatin were diluted prior to analysis to an assay 1:4. The maximal intra-assay CV for IL-6 was 7.8% and the maximal inter-assay CV was 9.6%. Serum IGF-1 and FGF21 were also evaluated via a sandwich ELISA (R&D Systems, United States, catalog no DG100 and DF2100). Average intra-assay CV reported by the manufacturer was 4% for both. The detection sensitivity of IGF-1 and FGF21 was 0.056 ng mL^-1^ and 8.69 pg mL^-1^, respectively. The maximal inter-assay CV was 8.3% for IGF-1 and 10.9% for FGF21. Concentrations of serum irisin were determined using competitive enzyme immunoassay kits from Phoenix Pharmaceuticals Inc (catalog no EK 067-16). Intra-assay CV and inter-assay CV were 4–6 and 8–10%, respectively.

The serum concentrations of total cholesterol (TCH), high density lipoprotein cholesterol (HDL) and triglycerides (TG) were determined with the use of commercial kits based on enzymatic methods (Alpha Diagnostics, Poland). Glucose was assessed using *Cobos 6000analyzer*.

Quantification of serum leucine, isoleucine, valine and tryptophan was based on the ion-pair reversed phase high performance liquid chromatography combined with the tandem mass spectrometry IP-RP HPLC-MS/MS (TSQ Vantage Thermo Scientific). It was executed following the same procedure as in our previous study (Gmiat et al., 2018).

### Statistical Analysis

All measurements were compiled in a spreadsheet for the analysis of parallel-group trials and the effects were interpreted using magnitude-based inferences (Hopkins, 2006). All data were log-transformed to reduce bias arising from the non-uniformity of error; means of change scores in both groups, SDs of change scores and effects (variations of change in both the means and their confidence intervals: CI) were all back-transformed to percent units. Mean changes and effects were adjusted to the overall mean baseline values in both groups, by including the baseline value as a covariate in the analysis. Magnitudes of the effects were evaluated with the log-transformed data by standardizing the deviation of the baseline values. Threshold values for assessing magnitudes of standardized effects were 0.20, 0.60, 1.2 and 2.0 for small, moderate, large and very large effect, respectively. The uncertainty in the effects was expressed as 90% CI and as a probability of whether the true value of the effect was substantially positive (an increase) or negative (a decrease). For a non-clinical inference, the effect was deemed unclear if the CI overlapped thresholds for substantial increases and decreases (>5% in both cases as equivalent of 90% CI overlapping thresholds for a substantial increase and decrease). All other effects were deemed clear and were evaluated probabilistically. The scale for interpreting the probabilities was as follows: 25–75%, possible; 75–95%, likely; 95–99.5%, very likely; >99.5%, most likely.

## Results

All players completed our study with no injuries or adverse events being reported. Baseline anthropometric characteristics are presented in Table 2. Obtained results showed that no significant changes were recorded in body composition in response to training and coldness therapy.

**Table 2:** Participants’ body composition before and after the 2-week training program supported with the whole body cryostimulation protocol.

	Group	Baseline mean ± SD	Observed change mean ± SD (%)	Adjusted change^a^ mean ± SD (%)	Adjusted effect^b^	
					mean;CI (%)	lnference^c^
**Female**						
Body weight (kg)	CON	64.2 ± 9.7	-1 ± 2	-1 ± 2	2	Trivial
	CRY	64.0 ± 10.3	0 ± 1	0 ± 1	-2 to 5	
SMM (kg)	CON	27.3 ± 3.3	2 ± 4	2 ± 4	2	Trivial
	CRY	27.8 ± 3.7	8 ± 3	4 ± 3	4–9	
Body fat (%)	CON	22.9 ± 3.8	-8 ± 8	-8 ± 9	6	Small
	CRY	21.6+4.0	-4 ± 9	-2 ± 10	-8 to 21	
**Male**						
Body weight (kg)	CON	79.9 ± 8.1	-1 ± 1	-1 ± 1	0	Trivial
	CRY	79.7+8.1	-1 ± 1	-1 ± 1	-2 to 2	
SMM (kg)	CON	40.5 ± 2.6	0 ± 1	0 ± 1	1	Trivial
	CRY	40.8 ± 3.0	1 ± 2	1 ± 2	-2 to 5	
Body fat (%)	CON	10.9 ± 5.1	-3 ± 12	-2 + 14	-5	Trivial
	CRY	10.6 ± 4.5	-8 ± 11	-7 + 13	-23 to 19	

*SMM, skeletal muscle mass; CON, control; CRY, cryotherapy. CI, 90% confidence interval. All data are percentages, with the exception of baseline values express measurement units. ^a^Adjusted to overall mean of in baseline CON and CRY group. ^b^Adjusted mean change of CRY group minus adjusted mean CON group. ^c^Magnitude thresholds (for difference in means divided by SD of CRY group): <0.20, trivial; 0.20–0.59, small; 0.60–1.19, moderate*.

### The Effect of the Applied Training Program and Coldness Therapy/Passive Recovery Procedures on Physical Performance

Results obtained in volleyball tasks indicated that the training program applied with either of the recovery procedures did not affect significant participants’ physical performance. However, most of the measured parameters reflected attenuation of its performance. Changes recorded in the concentration test results were accompanied by changes in the serve accuracy test results. Given differing norms for performance in physical activity tests between men and women, we opted to present data according to participants’ sex. Still, when comparing the CRY and CON groups, the trend of change proved to be similar. In the CRY group, women achieved slightly better results in the concentration and serve accuracy tests than females in the CON group, whereas men exhibited poorer performance with the deterioration of the serve accuracy recorded as a statistically significant change. Changes in motor abilities and concentration test are shown in Table 3.

**Table 3:** Changes in motor abilities and concentration test after the 2 week training program combined with two different recovery methods (cryostimulation or passive rest).

	Group	Baseline mean ± SD	Observed change mean ± SD (%)	Adjusted change^a^ mean ± SD (%)	Adjusted effect^b^	
					mean;CI (%)	Inference^c^
**Female**
CT 1 (points)	CON	19.7 ± 8.0	-14 ± 86	-14 ± 96	14	Small
	CRY	20.0 ± 8.0	-3 ± 76	-2 ± 55	-44 to 130	
CT 2 (points)	CON	19.7 ± 8.1	5 ± 28	10 ± 11	21	Small
	CRY	16.5 ± 6.6	46 ± 51	33 ± 23	-14 to 70	
SAT test (points)	CON	30.3 ± 6.5	-9 ± 110	-15 ± 122	6	Small
	CRY	36.5 ± 11.1	-10 ± 2	-10 ± 4	-50 to 121	
CMJ 1 (cm)	CON	35.4 ± 4.2	2 ± 9	2 ± 10	0	Trivial
	CRY	31.5 ± 5.3	7 ± 16	2 ± 14	-17 to 22	
CMJ 1 (W⋅kg^-1^)	CON	46.9 ± 4.6	-25 ± 7	-25 ± 8	30	**Large ↑^∗∗∗∗^**
	CRY	55.3 ± 3.4	-3 ± 6	-3 ± 7	17–45	
CMJ 2 (cm)	CON	33.1 ± 4.1	3 ± 10	3 ± 11	-2	Trivial
	CRY	31.7 ± 3.7	4 ± 12	2 ± 9	-13 to 11	
CMJ 2 (W⋅kg^-1^)	CON	63.2 ± 8.0	-12 ± 54	-6 ± 18	8	**Small ↑^∗^**
	CRY	48.7 ± 5.3	3 ± 10	2 ± 11	-5 to 22	
**Male**
CT 1 (points)	CON	18.5 ± 3.0	-2 ± 23	7 ± 24	6	Trivial
	CRY	14.8 ± 5.8	22 ± 23	14 ± 7	-20 to 42	
CT 2 (points)	CON	17.5 ± 2.5	8 ± 35	16 ± 34	-22	Moderate
	CRY	17.2 ± 6.5	-6 ± 88	-9 ± 85	-59 to 51	
SAT test (points)	CON	28.0 ± 9.0	16 ± 37	14 ± 13	-30	**Moderate ↓^∗∗∗^**
	CRY	36.0 ± 6.0	-9 ± 23	-21 ± 16	-44 to -13	
CMJ 1 (cm)	CON	53.4 ± 4.6	-6 ± 4	-6 ± 5	0	Trivial
	CRY	45.9 ± 3.2	2 ± 14	-6 ± 15	-22 to 28	
CMJ 1 (W⋅kg^-1^)	CON	61.0 ± 3.6	-18 ± 5	-19 ± 5	8	**Small ↑^∗∗^**
	CRY	55.3 ± 4.8	-5 ± 13	-12 ± 1	1–16	
CMJ 2 (cm)	CON	51.0 ± 6.0	0 ± 5	2 ± 4	-4	Small
	CRY	44.2 ± 4.2	7 ± 19	-2 ± 17	-24 to 21	
CMJ 2 (W⋅kg^-1^)	CON	59.0 ± 4.5	0 ± 3	2 ± 3	-3	Small
	CRY	52.2 ± 3.8	6 ± 12	-2 ± 10	-17 to 13	

*CT1, concentration test at rest; CT2, concentration test after interval; SAT, service accuracy test; CMJ 1, countermovement jump with arms on the hips; CMJ 2, countermovement jump with arms swing; CON, control; CRY, cryotherapy. CI, 90% confidence interval. All data are percentages, with the exception of baseline values expressed in measurement units. Inferences shown in bold are clear at the 98% level of confidence. ^*a*^Adjusted to overall mean of in baseline CON and CRY group. ^*b*^Adjusted mean change of CRY group minus adjusted mean CON group. ^*c*^Magnitude thresholds (for difference in means divided by SD of CRY group): <0.20, trivial; 0.20–0.59, small; 0.60–1.19, moderate; >1.20, large. ↑ – increase; ↓ – decrease. Asterisks indicate effects clear at the 5% level and likelihood that the true effect is substantial: ^∗^ – possible, ^∗∗^ – likely, ^∗∗∗^ – very likely, ^∗∗∗∗^ – most likely*.

Outcomes achieved in countermovement jumps with arms on the hips revealed significant shifts in maximal power. In both groups, the training program resulted in a drop of power achieved in this drill. Still, this decrease was much more pronounced in the CON group participants, who had been subject to a passive recovery procedure. The adjusted effect was large and most likely in female participants and small and likely in male subjects. We have not presented the data obtained through interval drills as comparing results between players proved difficult. Gates used for interval drills were lighting up in random sequences in each drill; thus, each athlete completed a different sequence of moves, covering a different distance. We have, therefore, only presented cognitive-concentration test results (Table 3).

### The Effect of the First Single Session of WBC

The single session of WBC did not alter significantly the concentration of irisin but induced a significant drop of IL-6 and BDNF levels in the CRY group. The adjusted effects for these changes ranged from small and likely for IL-6 to moderate and very likely for BDNF (Table 4). Interestingly, in CRY group, a pronounced drop of BDNF concentration (-44% ± 80) was noted in men, whereas in women, a significant increase (87% ± 183) was observed (data not shown).

**Table 4:** The effect of first exposure to whole body cryostimulation or passive rest on immunological response and amino acid profile among all volleyball athletes.

	Group	Baseline mean ± SD	Observed change mean ± SD (%)	Adjusted change^a^ mean ± SD (%)	Adjusted effect^b^	
					mean;CI (%)	Inference^c^
Irisin (ng⋅mL^-1^)	Control	9.3 ± 2.0	8 ± 40	6 ± 39	-7	Small
	Cryotherapy	9.7 ± 1.1	-5 ± 27	-2 ± 22%	-24 to 13	
IL-6 (pg⋅mL^-1^)	Control	1.1 ± 0.9	-38 ± 118	-37 ± 104%	-30	**Small ↓^∗∗^**
	Cryotherapy	1.0 ± 0.8	-55 ± 69	-56 ± 66	(57 to 13%	
BDNF (ng⋅mL^-1^)	Control	20.5 ± 6.4	31 ± 52	36 ± 51	-37	**Moderate ↓^∗∗^**
	Cryotherapy	20.9 ± 13.2	-4 ± 169	-14 ± 69	-57 to -7%	
FGF-21 (pg⋅mL(1)	Control	103.0 ± 124.7	-28 ± 101	-19 ± 92	(56%	**Moderate ↓^∗∗∗^**
	Cryotherapy	202.0 ± 147.6	-66 ± 43	-64 ± 43%	-71 to -34	
IL-15 (pg⋅mL^-1^)	Control	2.1 ± 0.8	-28 ± 41	-25 ± 30	(10%	Small
	Cryotherapy	1.6 ± 0.9	-29 ± 64	-32 ± 53	-32 to 18%	
Myostatin (ng⋅mL^-1^)	Control	27.9 ± 11.3	16 ± 14	17 ± 15	-6	Trivial
	Cryotherapy	33.8 ± 8.5	13 ± 21	10 ± 22	-19 to 10	
IGF-1 (pg⋅mL^-1^)	Control	188.8 ± 34.5	-5 ± 107	-6 ± 106	9	Small
	Cryotherapy	199.4 ± 83.7	2 ± 32	2 ± 25	-28 to 64	
Valine (μmol⋅L^-1^)	Control	100.9 ± 25.9	5 ± 50	8 ± 25	27	**Moderate ↑^∗∗^**
	Cryotherapy	98.3 ± 29.9	41 ± 59	37 ± 44	2–59	
Leucine (μmol⋅L^-1^)	Control	101.8 ± 26.5	32 ± 57	26 ± 58	-3	Trivial
	Cryotherapy	125.8 ± 43.3	12 ± 42	23 ± 26	-29 to 33	
Isoleucine (μmol⋅L^-1^)	Control	117.5 ± 62.2	33 ± 51	33 ± 44	0	Trivial
	Cryotherapy	111.5 ± 41.0	33 ± 32	33 ± 28	-20 to 25	
Tryptophan (μmol⋅L^-1^)	Control	40.9 ± 11.2	31 ± 31	26 ± 30	-2	Trivial
	Cryotherapy	49.1 ± 14.3	18 ± 28	24 ± 24	-18 to 18	

*CI, 90% confidence interval. All data are percentages, with the exception of baseline values expressed in measurement units. Inferences shown in bold are clear at the 98% level of confidence. ^*a*^Adjusted to overall mean of in baseline control and cryotherapy group. ^*b*^Adjusted mean change of Cryotherapy group minus adjusted mean Control group. ^*c*^Magnitude thresholds (for difference in means divided by SD of Cryotherapy group): <0.20, trivial; 0.20–0.59, small; 0.60–1.19, moderate; >1.20, large. ↑ – increase; ↓ – decrease. Asterisks indicate effects clear at the 5% level and likelihood that the true effect is substantial: ^∗^ – possible, ^∗∗^ – likely, ^∗∗∗^ – very likely, ^∗∗∗∗^ – most likely*.

The applied WBC protocol also affected the level of FGF21concentration. We recorded a significant drop in FGF21 1 h after the first, single WBC exposure. The adjusted effect was moderate and very likely (Table 4). We did not observe any significant changes in myostatin, IGF-1 or IL-15 in response to the first cryo-session. The amino acid profile also remained unchanged.

### The Effect of the Whole Training Program Supported by Whole Body Cryostimulation or Passive Rest

The applied nine sessions of WBC resulted in a significant increase of the BDNF concentration in the CRY group, while in the CON group the opposite tendency was noted. The adjusted effect was moderate and likely (Table 5). Moreover, the adjusted effect for the shift in the level of BDNF in women athletes amounted to 97% (effect large and very likely), while in men, only a 20% rise was observed. Thereby, the effect of first session of WBC was maintained after the whole period of intervention (data not shown). The applied training program did not cause the concentration of irisin to shift regardless of the recovery procedure applied. A slight decrease of irisin was noted in both groups. No relationship was recorded between the level of irisin and BDNF.

**Table 5:** The effect of nine sessions of whole body cryostimulation or passive rest on immunological response and amino acid profile among all athletes.

	Group	Baseline mean ± SD	Observed change mean ± SD (%)	Adjusted change^a^ mean ± SD (%)	Adjusted effect^b^	

					mean;CI (%)	Inference^c^
Irisin (ng⋅mL^-1^)	Control	9.3 ± 2.0	-6 ± 26	-6 ± 27	-2	Trivial
	Cryotherapy	9.7 ± 1.1	-11 ± 25	-8 ± 22	-17 to 15	
IL-6 (pg⋅mL^-1^)	Control	1.1 ± 0.9	-41 ± 71	-40 ± 44	32	Small
	Cryotherapy	1.0 ± 0.8	-18 ± 228	-21 ± 219	-38 to 180	
BDNF (ng⋅mL^-1^)	Control	20.5 ± 6.4	-32 ± 74	-33 ± 78	113	**Large ↑^∗∗∗^**
	Cryotherapy	20.9 ± 13.2	57 ± 112	43 ± 30	38–230	
FGF-21 (pg⋅mL^-1^)	Control	103.0 ± 124.7	-16 ± 125	-22 ± 125	7	Trivial
	Cryotherapy	202.0 ± 147.6	-37 ± 101	-17 ± 86	-42 to 100	
IL-15 (pg⋅mL^-1^)	Control	2.1 ± 0.8	-8 ± 41	-6 ± 40	13	Small
	Cryotherapy	1.6 ± 0.9	4 ± 43	6 ± 40	-15 to 50	
Myostatin (ng⋅mL^-1^)	Control	27.9 ± 11.3	15 ± 32	13 ± 33	-5	Trivial
	Cryotherapy	33.8 ± 8.5	4 ± 28	8 ± 28	-24 to 20	
IGF-1 (pg⋅mL^-1^)	Control	188.8 ± 34.5	-22 ± 22	-22 ± 23	45	**Large ↑^∗∗∗^**
	Cryotherapy	199.4 ± 83.7	14 ± 50	14 ± 35	17–79	
Valine (μmol⋅L^-1^)	Control	100.9 ± 25.9	3 ± 61	10 ± 36	-18	**Moderate ↓^∗∗^**
	Cryotherapy	98.3 ± 29.9	-7 ± 35	-10 ± 24	-34 to 1	
Leucine (μmol⋅L^-1^)	Control	101.8 ± 26.5	2 ± 56	-8 ± 38	-13	Small
	Cryotherapy	125.8 ± 43.3	-28 ± 64	-20 ± 53	-38 to 23	
Isoleucine (μmol⋅L^-1^)	Control	117.5 ± 62.2	-7 ± 66	-6 ± 46	2	Trivial
	Cryotherapy	111.5 ± 41.0	-4 ± 64	-5 ± 69	-31 to 51	
Tryotophan (μmol⋅L^-1^)	Control	40.9 ± 11.2	10 ± 27	5 ± 27	-14	**Small ↓^∗∗^**
	Cryotherapy	49.1 ± 14.3	-20 ± 41	-10 ± 23	-29 to 4	

*CI, 90% confidence interval. All data are percentages, with the exception of baseline values expressed in measurement units. Inferences shown in bold are clear at the 98% level of confidence. ^*a*^Adjusted to overall mean of in baseline control and cryotherapy group. ^*b*^Adjusted mean change of Cryotherapy group minus adjusted mean Control group. ^*c*^Magnitude thresholds (for difference in means divided by SD of Cryotherapy group): <0.20, trivial; 0.20–0.59, small; 0.60–1.19, moderate; >1.20, large. ↑ – increase; ↓ – decrease. Asterisks indicate effects clear at the 5% level and likelihood that the true effect is substantial: ^∗^ – possible, ^∗∗^ – likely, ^∗∗∗^ – very likely, ^∗∗∗∗^ – most likely*.

The training program supported by WBC caused a rise of IGF-1 concentration, whereas in the CON group, a 22% drop was recorded. The adjusted effect for those changes was large and very likely. The intervention induced the drop of serum concentration of IL-15 in the CON group, yet caused the rise in the CRY group (Table 5). Still, when sex-dependent differences were considered, the level of IL-15 was found to have significant increased in men in the CRY group. The adjusted effect was moderate and very likely (data not shown in table). Interestingly, we have observed an inverse correlation between irisin and myostatin before and after the last session of WBC (*r* = -0.40 and *r* = -0.42, respectively). The observed changes were not statistically significant, yet in the CON group, a similar relationship had an opposite tendency and became more pronounced and significant (*r* = 0.66 and *r* = -0.72, respectively; *p* = 0.05 at the same time-points of blood collection).

Together with growth factors, we have evaluated the influence of training supported by two different recovery procedures on the amino acid profile. The combination of volleyball training and WBC protocol resulted in a significant drop of valine and tryptophan. The adjusted effect for these changes was moderate small and likely, respectively. The remaining amino acids assessed were not affected (Table 5).

Noteworthily, in the CRY group, the resting glucose level decreased significantly after the whole intervention. The adjusted change was equal -6% in the CON group, whereas in the CRY group, the shift was twofold- equal -12%. Alternations in glucose were not depended on sex. In addition, training supported by WBC induced changes in the lipid profile. The levels of HDL and TC dropped in the CON group, while in the CRY group, an increase of the level of HDL and a significant drop of the level of TC were noted. The adjusted effect for these shifts was trivial and possible for HDL and small and likely for TC (Table 6).

**Table 6:** Changes in glucose and lipid profile in response to 2 weeks of specific volleyball training supported by different recovery procedures.

	Group	Baseline mean ± SD	Observed change mean ± SD (%)	Adjusted change^a^ mean ± SD (%)	Adjusted effect^b^	
					mean;CI (%)	Inference^c^
TCH (mg⋅dL^-1^)	Control	166.3 ± 20.3	-5 ± 5	-5 ± 5	-7	**Small ↓^∗∗^**
	Cryotherapy	165.5 ± 30.3	-9 ± 16	-11 ± 12	-13 to 0	
HDL (mg⋅dL^-1^)	Control	75.2 ± 22.4	-3 ± 9	-4 ± 9	5	**Trivial ↑^∗^**
	Cryotherapy	56.4 ± 11.6	0 ± 12	1 ± 13	-5 to 17	
LDL (mg⋅dL^-1^)	Control	76.0 ± 19.2	-5 ± 13	-6 ± 12	0	Trivial
	Cryotherapy	89.4 ± 26.5	-8 ± 28	-6 ± 19	-12 to 13	
TG (mg⋅dL^-1^)	Control	75.2 ± 21.8	-20 ± 16	-22 ± 15	-4	Trivial
	Cryotherapy	98.7 ± 55.4	-30 ± 79	-24 ± 40	-23 to 21	
Glucose (mg dL^-1^)	Control	87.3 ± 5.7	-4 ± 8	-6 ± 5	-6	**Small ↓^∗∗∗^**
	Cryotherapy	91.0 ± 5.1	-12 ± 5	-12 ± 5	-10 to -2	

*TCH, total cholesterol; HDL, high density lipoprotein; LDL, low density lipoprotein; TG, triglyceride level. CI, 90% confidence interval. All data are percentages, with the exception of baseline values expressed in measurement units. Inferences shown in bold are clear at the 98% level of confidence. ^*a*^Adjusted to overall mean of in baseline control and cryotherapy group. ^*b*^Adjusted mean change of Cryotherapy group minus adjusted mean Control group. ^*c*^Magnitude thresholds (for difference in means divided by SD of Cryotherapy group): <0.20, trivial; 0.20–0.59, small; ↑ – increase; ↓ – decrease. Asterisks indicate effects clear at the 5% level and likelihood that the true effect is substantial: ^∗^ – possible, ^∗∗^ – likely, ^∗∗∗^ – very likely*.

## Discussion

This study demonstrates that specific volleyball training supported by WBC caused a significant increase of the levels of growth factors like IGF-1 and BDNF. Still, physical performance became attenuated in response to the intervention, though the extent of the drop was smaller in the CRY group. The concentration of BDNF is known to grow in response to a single session of exercise as well as regular training. This neurotrophic factor improves cognitive functions; it can also act as a modifying factor enhancing glucose and fat uptake (Huh, 2018). Although in our group of participants a small deterioration of motor abilities was recorded, the volleyball training applied in conjunction of WBC resulted in a significant elevation of BDNF concentration. With the range of change more pronounced among women, it can explain the less pronounced deterioration of the serve accuracy as well as better results obtained in the concentration test in comparison to the CON group. Based on reports showing that extremely low temperatures cause the concentration of irisin to grow (Dulian et al., 2015) as well as that the skeletal muscle-derived irisin may be the link between physical activity and reward-related processes and motivation (Zsuga et al., 2016), we have evaluated changes of irisin and BDNF levels among our athletes. We assumed that low amount of fat tissue among our athletes would induce a bigger thermogenic effect, muscle shivering and finally, lead to an increase of irisin. Contrary to our expectations, we have not observed any changes in irisin concentration as a result of the applied intervention. Moreover we evaluated the impact of applied procedure on second myokine: IL-6 concentration. In both groups a decline of this myokine was recorded. Still, obtained results, in opposite to previous observations (Ziemann et al., 2012) indicated on ambiguous changes, thus further investigations are needed. A recently published review has summarized the particular role of irisin in glucose homeostasis. Data show that the elevated concentration of irisin enhanced glucose and fatty acid uptake by 30–40%. This increase in glucose uptake resulted from the upregulation of glucose transporter type 4 (GLUT4) expression, without significant changes in the expression of genes encoding insulin receptors (Perakakis et al., 2017). Results obtained in the current study revealed that although no direct correlation between irisin and glucose was observed, the applied training program supported by WBC treatment led to a significant drop of glucose level and affected the lipid profile. These results correspond with data published in our previous paper, which reported reduced blood cholesterol in young college men only in response to cold treatment (the same schedule of procedure) (Ziemann et al., 2014). We have, thus, concluded that the positive impact of our intervention on glucose level was most likely the effect of cold exposures. In addition, we also observed the significant drop of FGF21 in response to the first session of WBC treatment and the same tendency (still slightly less) was maintained after whole intervention. This myokine is also regulated by coldness therapy (Lee et al., 2014). Lee et al. (2014) noted that a cold treatment like water-infused thermoblankets (from 27°C cooled to 18°C and cooled further by 2°C every 3 min until 12°C temperature was reached) was found to have stimulated irisin secretion and a drop of FGF21. A drop of the FGF21 concentration implies activation of the sympathetic nervous system in the response to cold treatment. Therefore, changes in its level, in our opinion, have contributed to a better glucose uptake at the end of the intervention.

Together with irisin, we have evaluated changes of myostatin in response to our intervention. To the best of our knowledge, there is no study showing the effect of exposure to cold temperature on myostatin serum level in humans. In our experiment, we did not register any significant shifts in myostatin concentration in response to a single or to 10 sessions of WBC. However, the range of changes was smaller in the CRY group. Interestingly, the higher the increase of irisin noted, the lower the level of myostatin was. It is also worth of noting that although these correlations were not significant in the CRY group, the tendency was opposite and significant in the CON group. It has been shown that not only strength training but also aerobic training lead to a decrease in the production of myostatin in rat muscles (Heinemeier et al., 2007), yet contradictory results have been reported for humans. Walker et al. (2004) have demonstrated a decrease in plasma myostatin after 10 weeks of resistance training. On the other hand, some studies have found that a single bout of resistance exercise as well as a long-term strength training can lead to an increase in serum myostatin level in young males (Willoughby, 2004).

The rise of BDNF concentration was accompanied by the significant increase of IGF-1. As a result, in the CRY group, volleyball motor abilities deteriorated to a smaller extent than in the CON group. The elevated level of IGF-1 could have positively influenced muscle hypertrophy. Another myokine, IL-15, increased slightly in the CRY group, whereas the tendency in the CON group was opposite. In the male part of the CRY group, the effect was statistically significant. Rinnov et al. (2014) have observed up-regulation of IL-15 in response to endurance training and reported data suggesting that increased production of IL-15 may be involved in mediating training-induced muscle adaptation through mitochondrial biogenesis. Also, data presented by Tamura et al. (2011) have revealed a rise of IL-15 in response to 30-min treadmill running at 70% HR (max). Finally, a recently published set of data has shown that serum IL-15 was increased ∼5.3-fold immediately after resistance exercise, contributing to myofibrillar protein synthesis (Perez-Lopez et al., 2018). The volleyball training program applied in our study enhanced muscle endurance strength, while relying on rather low resistance workload.

Any application of diverse coldness methods with the intention of supporting recovery processes, especially in conjunction with specific training, should precisely monitor different effects observed throughout the treatment due to possible attenuation of adaptation training process. Such effect was registered by Roberts et al. (2015), who have revealed that resistance training supported by CWI attenuated adaptive changes in response to exercise. On the other hand, athletes who underwent CWI (10 min at 8°C) together with resistance training exhibited greater expression of PGC-1α and angiogenic vascular endothelial growth factors (Joo et al., 2016). In our intervention, we implemented a training program focusing mainly on explosive strength. This program relied on low resistance and fast repetition to induce athletes’ explosive power. The timing of training sessions was very demanding, and their intensity was very similar to the preparatory camps organized before main tournaments. On the one hand, participants complained about fatigue; on the other hand, the subjective range of experienced fatigue was smaller in the CRY group. General weariness translated into poorer results recorded in volleyball drills performed at the end of the intervention, yet the magnitude of this drop was lower in the CRY group. A longer training period, e.g., 6 or 16 weeks (Verma et al., 2015), which would have allowed for some recovery breaks, could have potentially proven more effective in boosting explosive power, however, further research would be required to verify this observations. Still, our study has limitations that warrant mention. We have examined the influence of cryostimulation only on short term effects on athletes’ motor abilities. Future investigations should pursue studying long term outcomes of similar procedures. The same battery of tests should be executed >4 weeks after the training program combined with WBC or passive rest. Levels of growth factors would be also worth checking in order to establish whether cryostimulation can affect long term benefits in adaptation markers and hence, inhibit accumulated fatigue and reduced performance.

The additional aim of our study was to investigate the effect of the applied procedure on the amino acid profile. We decided to assess the concentration of branched amino acids according to their crucial role in muscle synthesis and tryptophan. We have observed that the tryptophan concentration dropped in response to training supported by WBC. The program combined with cryo-sessions led to an increase in the uptake of tryptophan in the CRY group, especially among women. This can provide an explanation for their better results in the serve accuracy and concentration tests compared to men. Kaluzna-Czaplinska et al. (2017) have elaborated extensively on tryptophan and its metabolites, emphasizing the role of tryptophan as a precursor of biologically active compounds such as serotonin and melatonin and showing that better uptake resulted in improved cognitive functions. Among other amino acids, we have noted a significant decrease of valine blood concentrations, however, this shift could have had a double meaning. On the one hand, just like other BCAAs, valine is associated with hyperglycaemia or insulin resistance (Lynch and Adams, 2014), thus, a decrease in valine could be associated with a boost of the overall metabolic profile—the change we recorded in our athletes. On the other hand, cryostimulation is known to be associated, at least as far as the first adaptation phase goes, with a mild haemolysis (Lombardi et al., 2013). Given that valine is essential for hematopoietic stem cell self-renewal (Taya et al., 2016), its mild decrease can be associated with an increased request from the bone marrow to restore haematopoiesis. The remaining branched amino acids exhibited the same tendency, with changes more pronounced in the CRY group. Although the effect was small, better uptake of those amino acids could have counteracted fatigue and deterioration of physical performance. As a result, in the CRY group physical capability was less affected than in the CON group.

Overall, our findings indicate that a specific volleyball training program induced fatigue, leading to a decline in the results achieved in the applied drill tests. Still, the magnitude of the drop was smaller in the CRY group compared to the CON group. At the same time, the applied protocol of recovery stimulated a higher increase of growth factors in the CRY group, which possibly counteracted further deterioration of physical performance. Our results characterize the response to the applied procedure as sex-dependent, however, the number of participants in our groups can be considered small. Therefore, we used a statistical model based on the effect size to validate our observations. Further research would be recommended to confirm our conclusions. To the best of our knowledge, most of the published papers up to date, evaluated the effect of WBC when associated to an exercise modality or middle intensity training, in particular endurance training. Our investigation considers the association of WBC with specific resistance-volleyball training and specific volleyball physical workload, what is rarely treated in literature.

## Ethics Statement

The experiment was approved by the Bioethical Committee of the Regional Medical Society in Gdańsk (KB-25/14).

## Author Contributions

JJ, KM, MK, KW, LR, and JS designed the study and performed the research. JJ, AB, GL, and EZ performed the research and wrote the paper. ER, GL, KW, and JS performed the research.

## Conflict of Interest Statement

The authors declare that the research was conducted in the absence of any commercial or financial relationships that could be construed as a potential conflict of interest.
